# Systematic Review of Complementary and Alternative Veterinary Medicine in Sport and Companion Animals: Extracorporeal Shockwave Therapy

**DOI:** 10.3390/ani12223124

**Published:** 2022-11-12

**Authors:** Anna Boström, Anna Bergh, Heli Hyytiäinen, Kjell Asplund

**Affiliations:** 1 Department of Equine and Small Animal Medicine, Faculty of Veterinary Medicine, University of Helsinki, P.O. Box 57, 00014 Helsinki, Finland; 2 Department of Clinical Sciences, Swedish University of Agricultural Sciences, SE 750 07 Uppsala, Sweden; 3 Department of Public Health and Clinical Medicine, Umeå University, SE 901 87 Umeå, Sweden

**Keywords:** extracorporeal shockwave therapy, radial shock-wave therapy, veterinary medicine, complementary and alternative veterinary medicine, companion animals, horse, dog, musculosketetal disorders

## Abstract

**Simple Summary:**

Extracorporeal shockwave therapy (ECSWT) is widely used in sport and companion animals to treat disorders affecting muscles, joints, and bones. Using a shockwave generator, a probe is applied to the skin over the affected area. Typically, there are one to three treatment sessions at 1- to 3-week intervals. The scientific basis for the treatment has been queried. This systematic review evaluated the scientific literature on shockwave therapy used in horses, dogs, and cats. The review revealed that only weak scientific evidence exists for favourable effects in conditions affecting bones, ligaments, tendons, and muscles in horses and dogs. No scientific article on the use of shockwave therapy in cats was available. Reasons for the weak scientific evidence were that studies were few, most involved only a small number of animals, many had methodological problems, or, when favourable results were reported, they were not replicated in independent studies. Thus, in sport and companion animals, the scientific evidence for the clinical effects of ECSWT in horses, dogs, and cats is limited. For a few indications, notably short-term pain relief, ligament ailments, and osteoarthritis, some results appear promising, warranting further exploration in high-quality studies.

**Abstract:**

Background: Extracorporeal shockwave therapy (ECSWT) is increasingly used to treat different types of musculoskeletal conditions in sport and companion animals. To explore the scientific basis for the treatment, we conducted a systematic review of the literature on ECSWT used in horses, dogs, and cats. Methods: Relevant articles published in 1980–2020 were identified from three major databases. Each article was assessed for risk of bias. Results: The review identified 27 relevant articles on the effects of ECSWT in horses, nine in dogs, but none in cats. Typically, ECSWT involved one to three treatment sessions at 1- to 3-week intervals. We identified studies on bone mass and bone healing, wound healing, navicular disease, ligament injury, desmitis, sesamoiditis, tendon injury, osteoarthritis, and short-term analgesic effects. Common to all indications was that the scientific evidence was very limited. For each separate indication, there were relatively few studies, many of which had methodological flaws. Where favorable results were reported, they were usually not replicated in independent studies. A few encouraging results were found. Conclusions: In sport and companion animals, the scientific evidence for clinical effects of ECSWT in horses, dogs, and cats is limited. For some applications, notably short-term pain relief, ligament ailments, and osteoarthritis, the results seem promising and warrant further exploration in high-quality studies.

## 1. Introduction

Extracorporeal shockwave therapy (ECSWT) is increasingly used to treat musculoskeletal conditions in both animals and humans [1,2]. In ECSWT, shockwaves are distributed to the tissue via an applicator directly applied to the affected area [3]. There are several variants of ECSWT, including focused and radial shockwaves and high-energy as well as low-energy treatments [3].

The technique was originally developed to dissolve urinary tract stones in humans [4]. Early in this development, it was noted that ECSWT for kidney stones appeared to increase bone density in nearby bones [4]. This subsequently led to the use of ECSWT in a widening variety of musculoskeletal indications [1]. Veterinarians as well as complementary and alternative practitioners treating animals now use the technique to treat, for instance, horses with tendon and ligament injuries, spinal problems, and arthritis [1]. 

The mechanism of action is, however, not completely known, and the scientific evidence for any benefits of ECSWT in animals has been questioned [1]. To the best of our knowledge, no systematic literature review on the effects of ECSWT for clinical indications in veterinary medicine has been published. The aim of this work was to systematically review the scientific literature on ECSWT in horses, dogs, and cats, with an emphasis on the effects on outcomes of clinical relevance. 

## 2. Materials and Methods

This systematic review is part of a broader review project, including a large number of complementary and alternative veterinary medicine (CAVM) therapies used in sport and companion animals, reported in this special issue of Animals. In August 2020, professional librarians searched the literature of the Web of Science Core Collection, the Center for Agriculture and Bioscience International (CABI) database, and PubMed (1980–2020). The basic literature search terms, common to all reviews, were dog OR cat OR horse, AND veterinary medicine OR veterinarian, AND therapy* OR treatment* [5]. For the present review, the specific search terms were extracorporeal shockwave therapy, radial shockwave therapy, pulsed shockwave therapy, and electrotherapy. Two authors (HH, ABo) undertook the selection and review of the articles.

### 2.1. Review Topic

Assessment of the scientific evidence for clinical efficacy of the use of ECSWT in sport and companion animals. 

### 2.2. General Inclusion and Exclusion Criteria

The inclusion criteria were that the publication must be in a peer-reviewed journal, be accessible by the authors through institutional access or internet search, and be a primary research publication. In the initial search stage, there were no restrictions regarding either country or language of publication. The study should describe the effects of ECSWT in the treatment of a single indication in horses, dogs, and cats. The studies should be randomized controlled trials (RCTs), other interventional studies, or observational studies. A therapeutic intervention was defined as an intervention intended to reduce the signs, severity, or duration of a clinical condition. Laboratory experimental studies were included only if they mimicked a clinical situation and/or a mechanism of action was investigated. Case series were included only if five or more subjects were reported. Because of risk of confounding, an exclusion criterion was any intervention that involved any type of treatment concomitant to ECSWT. 

### 2.3. Study Selection and Categorization

In the screening phase, we identified articles of possible relevance for the review. Screening was performed based on journal title, publication title, or abstract. Citations identified were imported into Endnote (X9.3.3, 2018) (Clarivate, London, Great Britain) and duplicates were removed. Two authors (ABo and HH) applied inclusion and exclusion criteria to all ECSWT publications. 

Articles identified in the screening phase were selected for full text evaluation. Articles not accessible from digital library resources were requested via the Swedish University of Agricultural Sciences library. If the full manuscript was not found following these steps but an abstract was available, categorization was carried out based on the abstract. 

For each study, the following key descriptive items were tabulated using templates modified from the Swedish Agency for Health Technology Assessment and Assessment of Social Services (SBU) [6]: first author, year of publication, study design, study population, intervention, control group, outcome, and relevance (external validity). 

Assessment of scientific quality in terms of risk of bias of each article was carried out in accordance with the Cochrane [7] and SBU [6] guidelines (Supplementary Material). The assessment was based on the following items: study design, statistical power, deviation from planned therapy, loss to follow-up, type of outcome assessment, and relevance. In observational studies, risk of confounding factors was also included in the assessment. To ensure consistency, prior to starting the literature review, three of the authors (KA, HH, AB) independently screened a random sample of articles; differences were discussed and resolved before reviewing all articles.

To summarize the body of evidence for each indication in which ECSWT was applied, we used the GRADE system [6,7]. It categorizes the certainty of evidence as ‘high’, ‘moderate’, ‘low’, or ‘very low’ by taking into account the following criteria of relevance for the present review: (a) risk of bias or other limitations in study design and implementation, (b) unexplained heterogeneity or inconsistency of results, and (c) imprecision of results (low statistical power). There are also domains that may increase the certainty of evidence: large effects, a dose-response gradient, and plausible confounding leading to the underestimation of effects. The writing of the paper has been conducted following the PRISMA 2022 check list. The study has not been registered in PROSPERO since it is not for human health.

## 3. Results

### 3.1. Characteristics of the Literature

**Figure 1:**
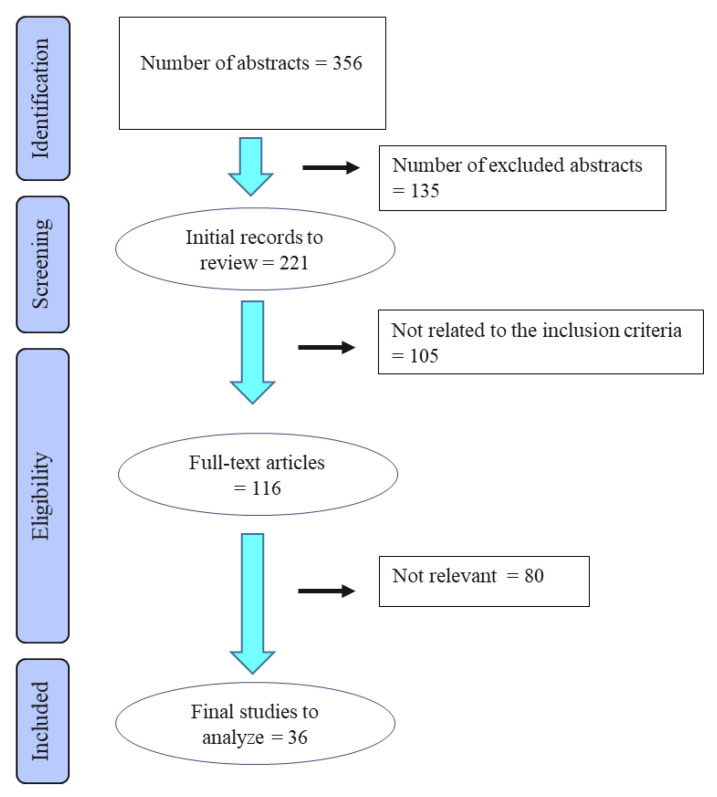
Flow diagram of the stages of the selection process used for identification of studies eligible for final analysis.

Of a total of 116 articles screened for possible relevance in this review, 36 studies fulfilled the inclusion criteria (see Figure 1).

**Figure 2:**
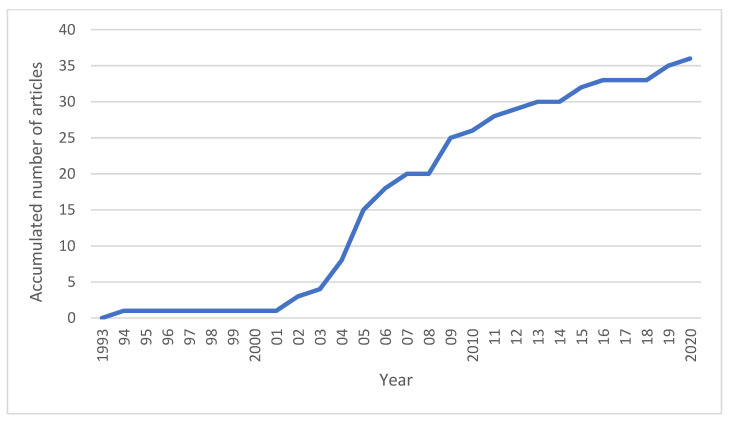
Accumulated number of articles fulfilling the inclusion criteria for systematic reviews of studies evaluating ECSWT in sport and companion animals published during 1993–2020.

Of the included studies, 27 were performed in horses (one of which reported on three different clinical conditions) [8], nine in dogs, and none in cats. The first article fulfilling our inclusion criteria was published in 1994 [9]. During the years 2004–2009, there was a surge in the number of articles, with more than half of all articles being published during this period (Figure 2). Since then, there has been a modest growth of literature evaluating ECSWT, with zero to two articles published each year. Sixteen of the included articles reported on follow-up of ECSWT in a group of at least five animals (clinical cohort study); of these, four had a control group and 12 did not. Most of the clinical cohort studies had a high risk of bias. Seven publications reported on RCTs, of which three studies had low and four had a moderate risk of bias. Another 11 were experimental studies in sound horses; seven of these studies had a low risk of bias.

Eighteen of the publications were from the USA, five from Germany, three each from Austria, Brazil, and Canada, and two each from Switzerland and the UK. Key characteristics of the ECSWT treatment reported in the publications (number of sessions, treatment intervals, number of pulses per ECSWT session, energy density (or similar)) are given in Table 1 and Table 2. The tables also summarize information on study population, controls, outcome variables, main results, and study risk of bias.

### 3.2. Horses

Detailed information on the equine studies’ design, study population, dosages (mJ/mm^2^), outcomes, results, and study risk of bias are presented in Table 1.

#### 3.2.1. Bone Mass

*Experimental studies.* In a non-randomized trial with a moderate risk of bias, the ECSWT effects (three sessions with 3-week intervals) on the elasticity of metacarpal bone tissue and bone mass was investigated in 10 healthy thoroughbred racehorses [10]. Ten non-treated horses constituted the control group. During a 72-day follow-up a statistically significant temporary decrease in bone mass was observed.

In another experimental study with low risk of bias, six healthy horses were subjected to ECSWT (two sessions with a 16-day interval) at two locations in the forelimb: the origin of the suspensory ligament and the fourth metacarpal bone [11]. Using scintigraphy to measure radiopharmaceutical activity ratios, no significant differences in radiopharmaceutical activity ratios between the two groups were observed at the 19-day follow-up.

*Overall assessment of published studies.* Experimental studies in sound horses showed that the certainty of evidence (as assessed by GRADE, see Methods) for beneficial or detrimental effects of ECSWT on bone mass or radiopharmaceutical activity ratios were *very low* because of inconsistent results and low statistical power (imprecision).

#### 3.2.2. Wound Healing

*Experimental studies of induced wounds.* We identified three reports from two different experimental studies in which ECSWT was used to stimulate the healing of induced wounds in horses [12,13,14].

In a controlled experimental study with a low risk of bias, six healthy horses were treated with ECSWT; induced wounds in the contralateral limb were used as the control [14]. The results suggested that active treatment reduced the formation of granulation tissue and reduced signs of inflammation. However, ECSWT did not accelerate healing. 

In an enlarged study involving 14 horses with experimentally induced forelimb ulcers, the same research group compared growth factors in ECSWT-treated ulcers and non-treated ulcers in the contralateral forelimb [12]. ECSWT was associated with reduced TGF-β1 expression and increased IGF-1 expression, but there were no changes in another five investigated growth factors.

An experimental study with a low risk of bias evaluated the effects of ECSWT on the healing of experimentally-induced metatarsal wounds in six horses [13]. Time to complete healing was compared with the healing of non-treated lesions in the same horses. ECSWT was applied at one-week intervals until the wound had healed. Mean healing time was significantly shorter in wounds treated by ECSWT (74 days) than in control wounds (90 days). Analyses of biopsies failed to identify a mechanism of action for the improved healing.

*Overall assessment of published studies.* The certainty of evidence for beneficial effects in clinical practice was *low* because only experimental studies on the effects of ECSWT in induced wounds have been published and their statistical power was low (imprecision). However, two of the studies indicated beneficial effects.

#### 3.2.3. Navicular Disease

*Clinical cohort studies.* Four prospective cohort studies on navicular disease in horses were identified [8,15,16,17], only one of which had a control group [16]. The four studies were all assessed as having a high risk of bias.

A non-randomized study examined ECSWT given at two different sites during two sessions with a 4week interval in 42 horses with navicular disease: straight to the frog and to the bulb of the heel [15]. There was no control group. After a 6-week observation period, 47% of the horses treated against the frog were sound and 80% of the horses treated against the bulb of the heel were sound.

Byron et al. [17] examined radiographic changes in the navicular bone area with radiography and scintigraphy after a single ECSWT session and concluded that the therapy had no acute effect. According to the authors, analgesia rather than effects on local tissue metabolism can explain possible positive clinical effects observed in clinical practice. Another cohort study quantified lameness using force-plate analysis in nine horses with navicular disease [16]. No beneficial acute effect of ECSWT applied to the radicular bone area and the heel bulb was observed. This was in contrast to palmar digital local anesthetics, which significantly reduced lameness.

In a cohort study without a control group, assessed to have a high risk of bias, 12 horses with navicular disease received ECSWT (two to four sessions with 3- to 4-week intervals) [8]. At clinical follow-up examinations at 3 months, six horses were sound and at 6 months, five of these horses remained sound.

*Overall assessment of published studies.* The certainty of evidence for the beneficial effects of ECSWT in horses with navicular disease was *very low* because of inferior study design with a high risk of bias and heterogeneous results (inconsistency).

#### 3.2.4. Ligament Injury, Desmitis, and Sesamoiditis

*Experimental study.* In an experimental study with a low risk of bias, suspensory ligament desmitis was induced in both hind-limbs of 10 horses [18]. One of the limbs was then treated with ECSWT (three treatments at 3-week intervals); the other hindlimb served as a control. The active therapy appeared to stimulate the healing process as measured by both ultrasound and TGF expression in biopsies.

*Randomized controlled trial.* An RCT compared the effects of ECSWT (one to three treatments at 1-week intervals) and platelet-rich plasma in 96 horses with fore- or hind limb lameness localized to the proximal suspensory ligament [19]. Although both initial and short-term follow-ups were conducted blinded to treatment assignments, the trial was assessed as having a high risk of bias due to a high drop-out rate and a long-term follow-up that relied on phone calls instead of clinical examinations or diagnostic imaging. A higher proportion (26/34) of ECSWT-treated horses than horses treated with platelet-rich plasma (16/24) had returned to full work after one year (76% vs. 67%).

*Non-randomized clinical trial*. In a non-randomized study with a moderate risk of bias, Siedler et al. [20] assigned 47 horses with proximal suspensory desmitis to either two sessions of ECSWT with an approximately 10-day interval, or local injections of a mixture of local anesthetics, amino acids, homeopathic remedies, vitamin B, and heparin. At the 6-month follow-up of the 28 horses receiving ECSWT, at clinical examination 18 were assessed as healed and eight as improved. Of the 19 horses in the comparison group (mixture of medicines), 11 were assessed as healed and five as improved.

*Clinical cohort studies*. Crowe et al. [21] followed 64 horses treated with ECSWT (three treatments at 2-week intervals) for chronic or recurrent proximal suspensory desmitis. There were no controls, and the study was assessed to have a high risk of bias. At the 6-month follow-up, 52% of horses had returned to full work.

In a second cohort study with moderate risk of bias, 52 horses with chronic proximal suspensory desmitis were treated with ECSWT (3 sessions at 3-week intervals) [22]. No controls were used. At the 12-week follow-up, 80% with forelimb and 40% with hindlimb desmitis were assessed as sound. At 6 months, the proportions of sound horses were 53% and 41%, respectively.

In a non-randomized controlled cohort study with a high risk of bias, Löffeld et al. [23] followed 31 horses treated with ECSWT (one to six treatments) for chronic proximal suspensory desmitis. By 6 months after treatment, 71% of the ECSWT-treated horses had resumed full work. In the control group receiving conventional treatment, 50% had returned to full work. The difference between the two groups was statistically significant.

In a cohort study without a control group and assessed to have a high risk of bias, 34 horses with insertion desmopathy (including chronic proximal suspensory ligament desmitis) were treated with ECSWT (two to four sessions with 3-to 4-week intervals) [8]. At clinical follow-up examinations, 26 horses were reported to have improved at 3 months, and 27 had improved at late follow-up (up to 30 months).

In the same study, 10 horses with sesamoiditis were treated with ECSWT (two to four sessions with 3- to 4-week intervals) [8]. At clinical follow-up examinations, seven of the horses were reported to have improved at 3 months, six of which were also improved at the late follow-up (up to 30 months).

*Overall assessment of published studies.* The certainty of evidence for favorable effects of ECSWT in horses with ligament injury, desmitis, or sesamoiditis was *very low* because of the high risk of bias in most studies. However, the positive results of one RCT, one non-randomized trial, and two observational studies, despite having a high or moderate risk of bias, could warrant the high-quality evaluation of ECSWT in these conditions.

#### 3.2.5. Tendon Injury

*Experimental study.* In an experimental study with a high risk of bias, the effects of ECSWT (two sessions with a 6-week interval) on biochemical parameters, tenocyte metabolism, and histology of tendinous structures were evaluated in six healthy Shetland ponies [24,25]. The contralateral limb was used as a control. Three hours after the last treatment, glycosaminoglycan and protein synthesis was increased, but synthesis decreased 6 weeks after treatment. Histologically, a disorganization of the normal collagen structure was observed 3 h after ECSWT. Remnants of the first treatment were still visible after 6 weeks. The authors concluded that short-term stimulating effects of ECSWT might accelerate the initiation of the healing process in an injured tendon [25], but there were also early signs of disorganization of the tendon collagen network [24]. The long-term effects seemed less beneficial.

*Case series.* Hunter et al. [26] described a case series, with high risk of bias, involving eight racehorses with damage to the deep flexor tendon treated by ECSWT (single treatment). Five of the eight horses returned to successful racing.

*Overall assessment of published studies.* Scientific information on clinical effects of ECSWT on tendon injuries in horses is sparse. The certainty of evidence for beneficial effects was graded as *very low*.

#### 3.2.6. Osteoarthritis

*Experimental study.* In a randomized experimental study with a low risk of bias, Frisbie et al. [27] induced osteoarthritis in a carpal joint of 24 horses, of which eight were treated with ECSWT (two treatments with a 2-week interval), eight with a sham ECSWT probe, and eight with polysulfated glycosaminoglycan for 4 weeks. Clinical parameters were assessed by surgeons blinded to treatment assignments. At clinical examinations 70 days after the induction of osteoarthritis (56 days after onset of therapy), the degree of lameness was significantly lower in the ECSWT-treated group than in the two control groups. Examinations of synovial fluid, synovial membranes, cartilage, and subcortical bone were unable to identify any possible mechanism of the clinical effects of ECSWT [27]. However, changes in circulating biomarkers suggested that ECSWT caused some degree of bone remodeling [28].

*Clinical cohort study.* In a cohort study with a high risk of bias, Urhahne et al. [8] followed the clinical course of nine horses diagnosed with bone spavin and treated with ECSWT (two to four sessions with 3- to 4-week intervals). At clinical examination 3 months after the treatment, four horses were assessed as improved. At late follow-up (up to 30 months), three horses had improved compared with baseline.

In another cohort study with a high risk of bias, McCarroll [29] reported the outcome of 74 horses diagnosed with bone spavin and treated with a single session of ECSWT. At follow-up approximately 3 months after treatment, 59 of the horses were assessed to have reduced lameness. Compared with baseline, there were no radiological changes.

*Overall assessment of published studies.* A randomized study on experimentally induced arthritis, of uncertain relevance for clinical osteoarthritis, suggests the beneficial effects of ECSWT. In clinical cohort studies, certainty of evidence for beneficial effects of ECSWT in horses with osteoarthritis was *very low* because of the high risk of bias.

#### 3.2.7. Analgesic Effects

*Experimental studies.* Three experimental studies on the possible analgesic effects of ECSWT in healthy horses were identified [30,31,32]. One of the aims of the studies was to explore analgesia as an adverse effect, possibly increasing the risk of injuries during exercise.

Bolt et al. [30] investigated the effects of ECSWT on the conduction velocity of the medial and lateral palmar digital sensory nerves of the forelimb in six sound horses. The risk of bias in this study was assessed as moderate. The corresponding nerves on the contralateral side served as controls. After a single ECSWT session, reduced conduction velocity in the sensory nerves was observed at 3 and 7 days after treatment, with some effects remaining after 35 days.

In an experimental study with a high risk of bias, the same authors examined the acute cutaneous analgesic effects of ECSWT in the metacarpal region of 12 sound horses [31]. The opposite corresponding cutaneous area served as the control. No significant differences between intervention and control areas was observed.

In a third experimental study with a low risk of bias, 18 sound horses were randomly allocated to one of three groups: ECSWT with either 1000 or 2000 pulses or radial pressure wave treatment (2000 pulses) delivered by a pneumatic shockwave generator [32]. No significant changes in skin sensitivity were recorded in any of the groups during a 48-h observation period.

*Clinical cohort studies.* In 12 horses with thoracolumbar pain, the effects of ECSWT (three treatments with 2-week intervals) were assessed with a pressure algometer measuring mechanical nociceptive threshold (MNT) [33]. There was no control group, and the study was assessed as having a moderate risk of bias. The majority of the horses showed improvements in MNT during a 56-day follow-up. The multifidus muscle cross-sectional area, measured by ultrasonography, did not change.

In another clinical cohort study without controls and a high risk of bias, Alves et al. [34] treated 12 horses with back pain and thoracolumbar desmitis. The authors reported improvement in clinical lameness at 90 days after treatment and improvement in tissue echogenicity assessed by ultrasonography at 120 days after the last treatment session.

Based on gait analyses, an acute, short-term analgesic effect was demonstrated after a single ECSWT session in nine horses with lameness [35]. Depending on the outcome variable measured, the analgesic effect of ECSWT was similar or even inferior to local anesthesia.

*Overall assessment of published studies*. There is some, albeit inconsistent, evidence that ECSWT has short-term analgesic effects in horses. The certainty of evidence for these effects was graded as *low* because of heterogeneous results (inconsistency) and low statistical power in all studies (imprecision).

### 3.3. Dogs

Table 2 presents the study design, study population, dosages (mJ/mm^2^), outcomes, results, and study risk of bias for reports on dogs.

#### 3.3.1. Bone Healing

*Randomized controlled trials.* Three RCTs examining the effects of ECSWT on bone healing in dogs undergoing osteotomy for cranial cruciate ligament injury were identified [36,37,38].

In one of the RCTs with a moderate risk of bias, 42 dogs with cruciate ligament rupture that underwent tibial plateau levelling osteotomy (TPLO) were randomized into two groups: (1) ECSWT immediately after surgery and 2 weeks later or (2) no ECSWT [38]. Radiographs with blinded assessments and clinical scorings showed improved bone healing in ECSWT-treated dogs.

A similar RCT, assessed to have a low risk of bias, involved 39 dogs with cruciate ligament injury that underwent TPLO surgery [37]. Using radiographs and densiometry, no differences were observed in bone healing between dogs randomized to ECSWT immediately after surgery and four weeks postoperatively or no ECSWT. Blinding of assessment was not described in this study.

In a third RCT, also with a moderate risk of bias, 16 dogs undergoing TPLO were randomized to ECSWT immediately after surgery and 2 weeks later or TPLO only [36]. Eight weeks after surgery, improved peak vertical force (PVF) was observed in ECSWT-treated dogs, whereas no improvement was seen in the control group. However, pain scores did not differ between the two groups. Information on blinding was not provided.

In an RCT with a moderate risk of bias (low statistical power), 10 dogs with non-union fractures, of whom five received ECSWT and five non-treated dogs constituted a control group, were followed for 12 weeks using objective outcome measures, radiographs, and histology. Healing was observed in four of the five treated dogs versus one of the five non-treated dogs [9].

*Overall assessment of published studies.* Promising results of ECSWT on bone healing have been reported. However, in the RCT with the lowest risk of bias, no beneficial effects were observed. Further research regarding bone healing is warranted.

#### 3.3.2. Ligament Desmitis

*Randomized controlled trial.* An RCT with a low risk of bias included 30 dogs with patellar ligament desmitis after TPLO surgery [39]. Half of the dogs were randomized to two ECSWT sessions 4 and 6 weeks after TPLO, with untreated dogs serving as controls. At 6 and 8 weeks after surgery, the patellar tendon was significantly less thick in the dogs that received ECSWT, suggesting beneficial effects on patella desmitis. With blinded ultrasonography assessment, no significant differences emerged between the two experimental groups with regard to signs of ligament disruption and periligament edema.

*Overall assessment of published studies*. The certainty of evidence for beneficial effects of ECSWT in dogs with ligament desmitis was graded as *low* because only one RCT with limited statistical power is currently available. The results require replication.

#### 3.3.3. Tendon Injuries

*Retrospective clinical study.* In a retrospective study with a moderate risk of bias, 29 dogs with supraspinatus and infraspinatus tendinopathies were treated with ECSWT (one to three treatment sessions) [40]. At late follow-up (mean 95 weeks after the last treatment), the owners of 17 of the 20 dogs available for follow-up assessment reported good to excellent outcomes. Ten of the dogs were still on medications or nutraceuticals for shoulder problems.

*Overall assessment of published studies*. Only one retrospective study (inferior study design) is available. The certainty of evidence for beneficial effects of ECSWT in dogs with tendon injuries was therefore graded as *very low*.

#### 3.3.4. Osteoarthritis

*Randomized controlled study.* In a study with a low risk of bias, 14 dogs with stifle osteoarthritis were randomly assigned to ECSWT (three treatments with 3-week intervals) or no ECSWT [41]. During a 98-day observation period, the ECSWT-treated dogs showed little change in kinetic force plate analysis and goniometry (not statistically significant), whereas sham-treated dogs worsened significantly. Symptoms reported by the dog owners, blinded to treatment groups, at follow-up did not differ between the two groups.

*Clinical cohort studies.* In a non-randomized study with a high risk of bias, Mueller et al. [42] investigated the effects of ECSWT (weekly treatments for 3 weeks) in 18 dogs with hip osteoarthritis relative to a control group of eight osteoarthritic dogs left untreated. At the six months follow-up, the authors reported an improvement in the lameness in dogs treated with ECSWT. In untreated dogs, no improvement was observed.

In another report from the same research group, 12 dogs with osteoarthritis of the elbow were treated with ECSWT weekly for 3 weeks [43]. There was no control group, and the study was assessed to have a high risk of bias. Over a 4-week observation period after the last treatment, improved gait was reported.

*Overall assessment of published studies.* The certainty of evidence for beneficial effects of ECSWT in dogs with osteoarthritis was graded as *low*; only one RCT with limited statistical power is available, and two observational studies have a high risk of bias. The somewhat encouraging results require replication.

### 3.4. Cats

No relevant study in cats was identified.

## 4. Discussion

ECSWT has been evaluated in seven indications in horses and in four indications in dogs; the scientific evidence was not strong enough in any of these to support the clinical effects of ECSWT. For a few indications, this review shows results that warrant further studies of the effects of ECSWT.

Of the 36 studies reviewed, 10 met the criteria for low risk of bias. Many of the studies were observational without a control group (or an inadequate control group). The extent to which the reported beneficial effects of ECSWT can be distinguished from spontaneous recovery is therefore difficult to assess. Of observational or interventional studies with a control group, few were large enough to have sufficient statistical power to detect significant differences between the groups. With many small studies, the risk of a type I error (a false-positive finding) in any of them increases. The results of our systematic literature review are in line with a recent non-systematic literature review focusing on ECSWT in the treatment of tendon and ligament injuries in horses [3]. That review found favorable effects of ECSWT in multiple musculoskeletal conditions but noted the poor quality of the experimental design in many studies.

There are likely several reasons why high-quality studies of ECSWT have seldom been performed in sport and companion animals. One possible explanation is that ECSWT is already accepted as an effective treatment method in veterinary medicine based on clinical experiences and the positive effects reported. Furthermore, evaluation of the study design of many of the studies covered by our review indicates that there is limited experience on how to conduct research of sufficient quality to provide justification for the research and veterinary practice community to accept or discard the therapy.

For many methods used in CAVM, possibly also including ECSWT, the incentives to improve the scientific evidence may be limited if there is an animal owner-driven market for the method; it seems likely that few CAVM customers ask for scientific documentation. Furthermore, CAVM therapists may be sceptical about the design of academic studies. In the human complementary and alternative medicine community, there is widespread criticism of randomized controlled studies as the pivotal high-quality standard to evaluate the effects of a therapy (e.g., [44]).

ECSWT is distinguished from many other CAVM therapies in that it is well-defined and, as our review shows, the way it is administered is relatively uniform (although the number of treatment sessions varies). In contrast to CAVM therapies like homeopathy, crystal therapy, or healing, ECSWT is based on conventional explanatory models generally accepted by the scientific community. The limited information available on possible mechanisms of action of ECSWT in animals with musculoskeletal problems suggests early stimulating effects on glycosaminoglycan and protein syntheses [24] and some modifying effects on growth factors in wounds [12]. In wounds, there are also some limited results indicating that ECSWT may reduce the formation of granulation tissue and inflammation in induced wounds in horses [14]. Studies of ECSWT effects in human musculoskeletal tissue have shown similar results but also additional possible mechanisms: neovascularization, anti-inflammatory effects, and the promotion of stem cell proliferation [45]. In the studies covered by our review, it should be noted that changes in various tissue biomarkers were not always accompanied by measurable clinical improvement, and that there are studies failing to show any mechanism of action despite extensive laboratory investigations.

In comparison with other CAVM methods, most of the ECSWT treatments, at least in Scandinavia, are given at veterinary practices, and this is preceded by a diagnostic work-up conducted by a veterinarian. In addition, ECSWT often requires sedation of the animal, which in many countries limits the use of this method to veterinarians.

No major long-term adverse effects of ECSWT have been reported in the articles included in the present review. However, an experimental study in healthy horses reported contradictory results on bone mass [10]. There has also been a concern that analgesic effects (for which there is some, albeit inconsistent, evidence) may increase the risk of injuries during exercise. An underlying, subclinical injury may be masked by the possible analgesic effects from the ECSWT treatment, allowing exercise and training to continue although tissues would require rest to avoid injury.

For none of the indications was the scientific evidence for ECSWT strong enough to include this therapy in routine veterinary practice; the studies have been negative, or they have been of insufficient quality, or the results of similar studies have been contradictory. For some indications, however, some beneficial results were reported, warranting further exploration in high-quality studies. One such group of indications is ligament injury and desmitis, where some encouraging results have been reported in both horses and dogs. Other indications worthy of further exploration are wound healing in horses and osteoarthritis in dogs. Short- and long-term analgesic effects of ECSWT also warrant investigation.

The main strength of our review is that this is, to the best of our knowledge, the first systematic literature review of the effects of ECSWT in sport and companion animals. We used established methods for the literature search, a selection of relevant articles, and extracting of information from the articles. Risk of bias in individual articles was based on templates developed by the Cochrane Collaboration [7] and the Swedish Agency for Health Technology Assessment and Assessment of Social Services (SBU) [6], and the certainty of evidence was assessed by the well-established GRADE system [6,7]. Professional librarians performed the literature search, and the search terms were selected after a pilot literature search. The broad search strategy had low specificity; only 36 of 116 articles initially identified by title and/or abstract (31.0%) eventually fulfilled the inclusion criteria.

A limitation for drawing conclusions on the treatment effects of ECSWT in sport and companion animals is the vast heterogeneity of the studies included in the review. This heterogeneity concerns study design, use of controls, statistical power, outcome measurements, and time to follow-up. The large share of low-quality studies with a high risk of bias is problematic but constitutes in itself a basis for the overall assessment of the present strength of scientific evidence. Because of the heterogeneity between the studies and since the number of studies was low for each species/indication combination, pooled statistical analysis with meta-analysis was not feasible. A further limitation is that in order to avoid confounding, no studies on the combination of ECSWT with other concomitant therapies were included.

As the aim of the literature review was to evaluate the scientific literature in sport and companion animals, the search was restricted to horses, dogs, and cats. Had more species had been included, additional publications could possibly have been identified, extending the information provided.

Researchers considering further studies of ECSWT in animals should discuss the results and how they can be interpreted from the perspective of previous studies and the working hypotheses. The findings and their implications should be discussed in the broadest context possible. Future research directions may also be highlighted.

## 5. Conclusions

The present systematic review has revealed significant gaps in scientific knowledge regarding the effects of ECSWT in horses and dogs. For the use of ECSWT in cats, no scientific articles were retrieved. For some of the musculoskeletal indications, at least some research documentation was available in horses and/or dogs. However, due to small sample sizes, lack of control groups, and other methodological limitations, few articles with a low risk of bias were identified. Where beneficial results were reported, they were seldom replicated in independent studies.

The large proportion of studies with a high risk of bias emphasizes the need for more high-quality research using precise methodologies to evaluate the potential therapeutic effects of ECSWT. For a few indications, notably wound healing, ligament injuries, desmitis, and osteoarthritis, some results seem interesting enough to warrant further exploration in high-quality observational or interventional studies. The possible analgesic effects of ECSWT may also be subject to more in-depth investigations. At present, the scientific evidence for the clinical effect of ECSWT in sport and companion animals is limited.

## Figures and Tables

**Table 1:** ECSWT in horses: Characteristics of studies included in the systematic review. Fibroblast growth factor-7 (FGF-7), Transforming growth factor-β1 (TGF-β1), Insulin-like growth factor-1 (IGF-1), Platelet derived growth factor-A (PDGF), Vascular endothelial growth factor-A (VEGF).

Main Author [Ref.] Publication Year Country	Study Design	Study Population	ECSWT: No. of Sessions No. of Pulses Energy Flux Density	Controls	Outcome Variables	Main Results	Study Risk of Bias
Bone mass							
Pyles [10] 2011 Brazil	Experimental controlled	20 sound thoroughbred horses (10 treated, 10 controls)	3 sessions 2000 pulses 0.15 mJ/mm^2^	Untreated	Bone elasticity, bone mass, risk of fracture (by ultrasound) during 72 days	Temporary moderate decrease in bone mass, statistically significant.	High
Ringer [11] 2005 Swizerland	Experimental controlled	6 sound horses	2 sessions 2000 pulses 0.15 mJ/mm^2^	Contralateral limb, untreated	Scintigraphy and thermography for 19 days	No statistically significant differences between the two groups at any timepoint.	Low
Wound healing							
Link [12] 2013 Canada	Experimental controlled	14 sound horses with induced forelimb wounds (6 of which were reported in Silveira [14])	6 sessions 900 pulses 0.11 mJ/mm^2^	Untreated wound in the same horse	Expression of growth factors (FGF-7, TGF-β1, IGF-1, PDGF, VEGF)	ECSWT associated with reduced TGF-β1 expression and increased IGF-1 expression at 35-day follow-up. No changes in other growth factors.	Low
Morgan [13] 2009 Canada	Experimental controlled	6 sound horses with induced metacarpal wounds	Weekly sessions until healing 500 pulses 0.11 mJ/mm^2^	Untreated wound in the same horse	Quantification of granulation tissue, radiographs, histology, immunohistology	Mean healing time significantly shorter in wounds treated by ECSWT (74 days) than in control wound (90 days). Analyses of biopsies failed to identify a mechanism of action.	Low
Silveira [14] 2010 Canada	Experimental controlled	6 sound horses with induced forelimb wounds (included in Link [12])	4 sessions 625 pulses No information on energy density	Untreated wound in the same horse	Blinded clinical assessments of wounds, histology, immuno-histochemistry	ECSWT associated with increased formation of granulation tissue and reduced signs of inflammation at the follow-up at 6–8 weeks. No effect on healing.	Low
Navicular disease							
Blum [15] 2005 Germany	Clinical cohort	43 horses with navicular disease	2 sessions 1200 pulses at either the frog or the bulb of the heel 0.15 mJ/mm^2^	None	Clinical examination and lameness score	At follow-up 6 weeks posttreatment, 47% treated at the frog and 80% treated at the bulb of the heel were assessed as sound.	High
Brown [16] 2005 Germany	Clinical cohort, controlled	9 horses with lameness	1 session 3000 pulses No information on energy density	Palmar digital local anesthetics	Force-plate measurements (ground reaction forces) for 7 days	In contrast to effects of local anaesthetics, ECSWT had no beneficial acute effects.	Moderate
Byron [17] 2009 USA	Clinical cohort	8 horses with unilateral forelimb lameness	3 sessions 3000 pulses No information on energy density	None	Radiographs, scintigraphy at follow-up at 14–20 days	No observed effect of ECSWT.	High
Urhahne [8] 2005 Germany	Clinical cohort	12 horses with navicular disease	2–4 sessions Total 1000–1500 pulses at 2–3 sites 0.15 mJ/mm^2^	None	Clinical examinations before treatment, early after treatment (<2 months), after 3 months, at 6 months, and at a later timepoint (up to 30 months).	Clinical improvement at both 3 months and late follow-up: 5/12 (42 %) “No notable adverse effects”.	High
Ligament injury, desmitis, and sesamoiditis							
Caminoto [18] 2005 Brazil	Experimental controlled	10 sound horses with experimentally induced suspensory ligament desmitis	3 sessions 1500 pulses 0.15 mJ/mm^2^	Untreated induced lesion in the same horse	Ultrasound, microscopy, immunohisto-chemistry followed for 14 weeks	ECSWT appeared to stimulate the healing process, as measured by both ultrasound and TGF-β1 expression in biopsies.	Low
Crowe [21] 2004 UK	Clinical cohort	64 horses with forelimb or hindlimb proximal suspensory ligament desmitis	3 sessions 2000 pulses No information on energy density	None	Return to work, lameness score, ultrasound at 6 months posttreatment	At follow-up, 52% of the horses had returned to full work.	High
Guinta [19] 2019 USA	Randomized controlled trial	96 horses with proximal suspensory desmitis	1–3 sessions 800 pulses 0.15 mJ/mm^2^	Platelet-rich plasma	Visual analog scale for work, lameness score, ultrasound	76% of ECSWT-treated horses had returned to full work one year posttreatment vs. 67% of horses treated with platelet-rich plasma.	Moderate (large number of horses lost to follow-up)
Lischer [22] 2006 UK	Clinical cohort	52 horses with chronic proximal suspensory desmitis	3 sessions 2000 pulses 0.15 mJ/mm^2^	None	Lameness score, flexion test, ultrasound at 12 weeks and 6 months	At 12 weeks: 80% with forelimb and 40% with hindlimb desmitis assessed as sound. At 5 months: 53% with forelimb and 41% with hindlimb desmitis assessed as sound.	High
Löffeld [23] 2002 Germany	Clinical cohort, controlled	31 horses with chronic proximal suspensory desmitis	1–6 sessions (mean 3) 2000 pulses 0.16 mJ/mm^2^	30 horses with chronic proximal suspensory desmitis, retrospective review of medical records	Lameness score followed for 6 months posttreatment	6 months posttreatment, 71% of ECSWT-treated horses had resumed full work vs. 50% of controls (statistically significant difference).	Moderate
Siedler [20] 2003 Austria	Clinical cohort, controlled	47 horses with proximal suspensory desmitis	2 sessions, ca. 10-day interval 2000 pulses 0.49 mJ/mm^2^	Injections of a mixture of local anesthetics, amino acids, two homeopathics, vitamin B, and heparin. A confounded control group (not included in this review)	Clinical examination and ultrasonography (m. interosseous medius) 6 months after ECSWT	Of 28 horses receiving ECSWT, 18 were assessed as healed and 8 as improved at 6-month follow-up. Of 19 horses in the comparison group (mixture of medicines), 11 were healed and 5 had improved. No statistical analyses.	Moderate
Urhahne [8] 2005 Germany	Clinical cohort	Horses with proximal suspensory ligament desmitis (n = 34) and sesamoiditis (n = 10)	2–4 sessions 1000–1500 pulses 0.15 mJ/mm^2^	None	Clinical examinations before treatment, early after treatment (<2 months), after 3 months, at 6 months, and at a later timepoint (up to 30 months)	Clinical improvement at 3 months: insertion desmopathy 26/34 (77%), sesamoiditis 7/10 (70%) Clinical improvement at late follow-up: insertion desmopathy 27/34 (79%), sesamoiditis 6/10 (60%) “No notable adverse effects”.	High
Tendon injury							
Bosch 2007 and 2009 [24,25] USA	Experimental controlled	6 sound Shetland ponies	2 sessions 1200 pulses 0.14 mJ/mm^2^	Untreated contralateral limb	Glycosaminoglycan and protein syntheses and histology of tendinous structures 3 h and 6 weeks after ECSWT	At 3 h, glycosaminoglycan and protein synthesis had increased and there was disorganization of the normal collagen structure. At 6 weeks posttreatment, glycosaminoglycan and protein synthesis was decreased and there was increased collagen disorganization.	Moderate
Hunter [26] 2004 USA	Case series	8 thoroughbred racehorses with superficial digital flexor tendonitis	2–3 sessions 1800–2000 pulses 0.13–0.15 mJ/mm^2^	None	Return to racing (follow-up 45 days to 8 months). Ultrasound: lesion size, lesion echogenicity, fiber alignment.	Five of 8 horses returned to racing, 2 horses were re-injured and 1 was retired.	High
Osteoarthritis							
Frisbie 2009 and Kawcak 2011 [27,28] USA	Experimental controlled	24 horses with induced carpal joint osteoarthritis (8 ECSWT-treated horses and two control groups)	2 sessions 1500–2000 pulses 0.14 mJ/mm^2^	Two control groups: sham ECSWT probe (8 horses), polysulfated glycosamino-glycan for 4 weeks (8 horses)	Lameness score, synovial fluid analysis, histology at 56 days posttreatment	Degree of lameness significantly lower in the ECSWT-treated group than in the two control groups. Examinations of synovial fluid, synovial membranes, cartilage, and subcortical bone did not identify any possible mechanism of the clinical effects of ECSWT. Changes in circulating biomarkers suggested some degree of bone remodeling.	Low
McCarroll [29] 2002 USA	Clinical cohort	74 adult horses with bone spavin	1 session 2000 pulses 0.89 mJ/mm^2^	None	Clinical examination and radiographs 11–16 weeks after treatment	Of 74 horses, 59 (80%) had reduced lameness at follow-up ca. 3 months after treatment. No changes at examination by radiography.	High
Urhahne [8] 2005 Germany	Clinical cohort	9 horses with spavin	2–4 sessions, 3–4-week intervals Total 1000–1500 pulses at 2–3 sites 0.15 mJ/mm^2^	None	Clinical examinations before treatment, early after treatment (<2 months), after 3 months, at 6 months, and at a later timepoint (up to 30 months)	Clinical improvement at 3 months: 4/9 horses (44 %). Clinical improvement at late follow-up: 3/9 horses (33 %). “No notable adverse effects”.	High
Analgesic effects							
Alves et al. [34] 2009 Brazil	Clinical cohort	10 horses with back pain and thoracolumbar desmitis	3 sessions, 21-day intervals. 500 pulses, 0.15 mJ/mm^2^, per lesion site	None	Ultrasonography and clinical tests: lameness evaluation, palpation at baseline and after the last treatment at 90 days	Results suggest the treatment to have positive effects on all horses, evaluated by clinical lameness scoring and ultrasound.	High
Bolt [30] 2004 a USA	Experimental controlled	6 sound horses	1 session 2000 pulses No information on energy density	Untreated contralateral forelimb	Conduction velocity of the medial and lateral palmar digital sensory nerves during 35 days posttreatment. Electron microscopy	Reduced conduction velocity in the sensory nerves at 3 and 7 days after treatment, with some effects remaining after 35 days. Transmission electron microscopy of treated nerves: disruption of the myelin sheath persisting at 35 days but no damage to Schwann bodies or axons.	Moderate
Bolt [31] 2004 b USA	Experimental controlled	12 sound horses	1 session 2000 pulses No information on energy density	Untreated contralateral forelimb	Cutaneous analgesia evaluated by withdrawal reflex latency during 48 h posttreatment	No significant differences between intervention and control areas.	Moderate
Dahlberg [35] 2006 USA	Clinical cohort	9 horses with unilateral forelimb lameness (6 with navicular syndrome, 3 with osteoarthritis)	1 session 2000 pulses 0.14 mJ/mm^2^	None	Lameness score, kinetic force plate analysis for 7 days posttreatment	Temporary reduction of lameness following ECSWT, duration 2 days.	High
Trager [33] 2020 USA	Clinical cohort	12 horses with physical evidence of back pain on examination	3 sessions 1500 pulses 0.13 mJ/mm^2^	None	Clinical assessment, pressure algometer recordings, ultrasound of multifidus muscle, radiography	Mechanical nociceptive threshold increased significantly at 35-day follow-up but not thereafter up to 56 days posttreatment. The multifidus muscle cross-sectional area did not change. Degree of radiographic change was not associated with response to treatment.	High
Waldern [32] 2005 Switzerland	Experimental	18 sound warm blood horses	1 session Three intervention groups: 1000 and 2000 pulses (0.15 mJ/mm^2^) and pneumatic shockwaves	None	Skin sensitivity tested during 48 h posttreatment	No significant changes in skin sensitivity in any of the groups during the 48-h observation period.	Low

**Table 2:** ECSWT in dogs: Characteristics of studies included in the systematic review.

Main Author (Ref.) Publication Year Country	Study Design	Study Population	ECSWT: No. of Sessions No. of Pulses Energy Flux Density	Controls	Outcome Variables	Main Results	Study Risk of Bias
Bone healing							
Barnes [37] 2015 USA	Randomized controlled trial	39 dogs with cranial cruciate ligament rupture undergoing tibial tuberosity advancement surgery with or without autogenous cancellous bone graft	2 sessions 1000 pulses 0.15 mJ/mm^2^	No ECSWT	Radiographs, densitometry	No difference in any outcome measure between ECSWT and non-ECSWT groups.	Low
Barnes [36] 2019 USA	Randomized controlled trial	16 dogs undergoing tibial plateau leveling osteotomy.	2 sessions 1000 pulses 0.15 mJ/mm^2^	No ECSWT	Pain score, joint range of motion, kinetic force plate analysis during 8-week follow-up	Peak vertical force and vertical impulse (reflecting weight bearing) increased faster and were significantly higher in the ECSWT-treated group at 8-week follow-up. Pain score and joint range of motion did not differ.	Moderate
Johannes [9] 1994 Germany	Randomized controlled trial	10 dogs with non-union fractures, 5 of whom received ECSWT	1–2 sessions 4000 pulses 0.54 mJ/mm^2^	No ECSWT	Radiographs at 12 weeks posttreatment	Authors reported significant difference in healing at follow-up (5/5 in ECSWT group, 1/5 in control group).	Moderate (low statistical power)
Kieves [38] 2015 USA	Randomized controlled trial	42 dogs undergoing tibial plateau leveling osteotomy.	2 sessions 1000 pulses 0.15 mJ/mm^2^	Sham ECSWT	Radiographs, 2 different scoring systems to quantify bone healing	Median healing score was significantly higher in ECSWT-treated dogs 8 weeks postoperatively.	Moderate
Ligament desmitis							
Gallagher [39] 2012 USA	Randomized controlled trial	30 dogs with unilateral patellar ligament desmitis after tibial plateau leveling osteotomy	2 sessions 600 pulses 0.15 mJ/mm^2^	No ECSWT	Radiography, ultrasound (blinded measurements)	Mean thickness of patellar ligament significantly lower in ECSWT-treated dogs at 6- and 8-week follow-ups. The difference was small (10–15%). No differences in signs of ligament disruption and periligament edema.	Low
Tendon injuries							
Leeman [40] 2016 USA	Retrospective(medical records), no controls	29 dogs diagnosed with supraspinatus or infraspinatus tendinopathies	4 sessions 750–1000 pulses 0.14–0.15 mJ/mm^2^	None	Owner assessment of outcome	At late follow-up (mean 95 weeks after the last treatment), 17 of the 20 owners available for follow-up assessment reported good to excellent outcome. Ten of the dogs were still on medications or nutraceuticals for shoulder problems.	High
Osteoarthritis							
Bockstahler [43] 2006 Austria	Clinical cohort	12 dogs with osteoarthritis of the elbow confirmed by radiography	3 sessions 1000 pulses 0.15 mJ/mm^2^	None	Kinetic force plate analysis before treatment and 4 weeks later	Significant improvement in gait-symmetry indices at the 4-week follow-up.	High
Dahlberg [41] 2005 USA	Randomized controlled trial	14 dogs with stifle osteoarthritis	3 sessions 3600 pulses 0.14 mJ/mm^2^	No ECSWT	Kinetic force plate analysis, goniometry, owner questionnaire (blinded)	During a 98-day observation period, ECSWT-treated dogs showed little change at kinetic force plate analysis and goniometry (not statistically significant), whereas sham-treated dogs worsened significantly. Symptoms reported by the dog owners did not differ between the two groups.	Low
Mueller [42] 2007 Austria	Clinical cohort, controlled	24 dogs with hip osteoarthritis	3 sessions 2000 pulses No information on energy density	No ECSWT	Kinetic force plate analysis before treatment and at 6 weeks, 3 months, and 6 months posttreatment	Significant improvements in vertical impulse and peak vertical force were observed 3 months after the treatment in ECSWT-treated dogs but not in the control group (but the groups were not balanced at onset).	High

## Supplementary Materials

Manual for assessment of risk of bias and relevance can be downloaded at: https://www.mdpi.com/article/10.3390/ani12223124/s1.

## References

[1] Chamberlain G.A., Colborne G.R. (2016). A review of the cellular and molecular effects of extracorporeal shockwave therapy. Vet. Comp. Orthop. Traumatol..

[2] MacKay A.V., McOnie R.C., Riddell L.P., Robinson K.A. (2020). Characterization of the use of shock wave therapy among equine veterinarians. Can. Vet. J..

[3] Yocom A.F., Bass L.D. (2019). Review of the application and efficacy of extracorporeal shockwave therapy in equine tendon and ligament injuries. Equine Vet. Educ..

[4] Hiller S.C., Ghani K.R. (2020). Frontiers of stone management. Curr. Opin. Urol..

[5] Bergh A., Lund I., Boström A., Hyytiäinen H., Asplund K. (2021). A systematic review of complementary and alternative veterinary medicine: “Miscellaneous therapies”. Animals.

[6] Statens Beredning för Medicinsk och Social Utvärdering (SBU) (2022). SBU:s Metodbok. https://www.sbu.se/sv/metod/sbus-metodbok/?pub=48286.

[7] Higgins J., Thomas J. (2021). Cochrane Handbook for Systematic Reviews of Interventions, Version 6.2.

[8] Urhahne P., Röcken M., Gerhards H. (2005). Eine klinische Studie zur Behandlung häufiger Erkrankungen des Bewegungsapparates des Pferdes mittels fokussierter extrakorporaler Stoßwellentherapie (ESWT). Pfederheilkunde.

[9] Johannes E.J., Kaulesar Sukul D.M.K.S., Matura E. (1994). High-energy shock waves for the treatment of nonunions: An experiment on dogs. J. Surg. Res..

[10] Pyles M.D., da Fonseca B.P.A., Machado V.M.V., Alves A.L.G. (2011). Determinação da densidade mineral e da elasticidade óssea após a aplicação de ondas de choque extracorpóreas no terceiro metacarpiano de equinos atletas. Braz. J. Vet. Tes. Anim. Sci..

[11] Ringer S.K., Lischer C.J., Ueltschi G. (2005). Assessment of scintigraphic and thermographic changes after focused extracorporeal shock wave therapy on the origin of the suspensory ligament and the fourth metatarsal bone in horses without lameness. Am. J. Vet. Res..

[12] Link K.A., Koenig J.B., Silveira A., Plattner B.L., Lillie B.N. (2013). Effect of unfocused extracorporeal shock wave therapy on growth factor gene expression in wounds and intact skin of horses. Am. J. Vet. Res..

[13] Morgan D.D., McClure S., Yaeger M.J., Schumacher J., Evans R.B. (2009). Effects of extracorporeal shock wave therapy on wounds of the distal portion of the limbs in horses. J. Am. Vet. Med. Assoc..

[14] Silveira A., Koenig J.B., Arroyo L.G., Trout D., Moens N.M., LaMarre J., Brooks A. (2010). Effects of unfocused extracorporeal shock wave therapy on healing of wounds of the distal portion of the forelimb in horses. Am. J. Vet. Res..

[15] Blum N., Kreling K., Öitzke L.-F. (2005). Der Einsatz der Extrakorporalen Stoßwellentherapie zur Behandlung des Podotrochlose-Syndroms. Pferdeheilkunde.

[16] Brown K.E., Nickels F.A., Caron J.P., Mullineaux D.R., Clayton H.M. (2005). Investigation of the immediate analgesic effects of extracorporeal shock wave therapy for treatment of navicular disease in horses. Vet. Surg..

[17] Byron C., Stewart A., Benson B., Tennent-Brown B., Foreman J. (2009). Effects of radial extracorporeal shock wave therapy on radiographic and scintigraphic outcomes in horses with palmar heel pain. Vet. Comp. Orthop. Traumatol..

[18] Caminoto E.H., Alves A.L., Amorim R.L., Thomassian A., Hussni C.A., Nicoletti J.L. (2005). Ultrastructural and immunocytochemical evaluation of the effects of extracorporeal shock wave treatment in the hind limbs of horses with experimentally induced suspensory ligament desmitis. Am. J. Vet. Res..

[19] Giunta K., Donnell J.R., Donnell A.R., Frisbie D.D. (2019). Prospective randomized comparison of platelet rich plasma to extracorporeal shockwave therapy for treatment of proximal suspensory pain in western performance horses. Res. Vet. Sci..

[20] Siedler C., Stanek C., Brems R. (2003). Proximal suspensory desmitis in the horse: Extracorporeal shock wave therapy compared to injections according to Dr. Muller-Wohlfahrt. A field study. Tierärtzl. Prax..

[21] Crowe O.M., Dyson S.J., Wright L.M., Schramme M.C., Smith R.K.W. (2004). Treatment of chronic or recurrent proximal suspensory desmitis using radial pressure wave therapy in the horse. Equine Vet. J..

[22] Lischer C.J., Ringer S.K., Schnewlin M., Imboden I., Fürst A., Stöckli M., Auer J. (2006). Treatment of chronic proximal suspensory desmitis in horses using focused electrohydraulic shockwave therapy. Schweiz. Arch. Tierheilk..

[23] Löffeld S., Boening K.-J., Weirkamp K., Stadlwer P. (2002). Radiale extrakorporale Stoßwellentherapie^®^ bei Pferden mit chronischer Insertionsdesmopathie am Fesselträgerursprung– eine kontrollierte Studie. Pferdeheilkunde.

[24] Bosch G., de Mos M., van Binsbergen R., van Schie H.T., van de Lest C.H., van Weeren P.R. (2009). The effect of focused extracorporeal shock wave therapy on collagen matrix and gene expression in normal tendons and ligaments. Equine Vet. J..

[25] Bosch G., Lin Y.L., van Schie H.T., van De Lest C.H., Barneveld A., van Weeren P.R. (2007). Effect of extracorporeal shock wave therapy on the biochemical composition and metabolic activity of tenocytes in normal tendinous structures in ponies. Equine Vet. J..

[26] Hunter J., McClure S.R., Merritt D.K., Reinertson E. (2004). Extracorporeal shockwave therapy for treatment of superficial digital flexor tendonitis in racing Thoroughbreds: 8 clinical cases. Vet. Comp. Orthop. Traumatol..

[27] Frisbie D.D., Kawcak C.E., McIlwraith C.W. (2009). Evaluation of the effect of extracorporeal shock wave treatment on experimentally induced osteoarthritis in middle carpal joints of horses. Am. J. Vet. Res..

[28] Kawcak C.E., Frisbie D.D., McIlwraith C.W. (2011). Effects of extracorporeal shock wave therapy and polysulfated glycosaminoglycan treatment on subchondral bone, serum biomarkers, and synovial fluid biomarkers in horses with induced osteoarthritis. Am. J. Vet. Res..

[29] McCarroll G.D., McClure S.R. (2002). Initial experiences with extracorporeal shock wave therapy for treatment of bone spavin in horses—Part II. Vet. Comp. Orthop. Traumatol..

[30] Bolt D.M., Burba D.J., Hubert J.D., Strain G.M., Hosgood G.L., Henk W.G., Cho D.Y. (2004). Determination of functional and morphologic changes in palmar digital nerves after nonfocused extracorporeal shock wave treatment in horses. Am. J. Vet. Res..

[31] Bolt D.M., Burba D.J., Hubert J.D., Pettifer G.R., Hosgood G.L. (2004). Evaluation of cutaneous analgesia after non-focused extracorporeal shock wave application over the 3rd metacarpal bone in horses. Can. J. Vet. Res..

[32] Waldern N.M., Weishaupt M.A., Imboden I., Wiestner T., Lischer C.J. (2005). Evaluation of skin sensitivity after shock wave treatment in horses. Am. J. Vet. Res..

[33] Trager I.R., Funk R.A., Clapp K.S., Dahlgren L.A., Werre S.L., Hodgson D.R., Pleasant R.S. (2020). Extracorporeal shockwave therapy raises mechanical nociceptive threshold in horses with thoracolumbar pain. Equine Vet. J..

[34] Alves A.L.G., Almeida da Fonseca B.P., Hussni C.A., Soares L.V. (2009). Tratamento da desmite supra e interespinhosa em equinos utilizando a terapia por ondas de choque extracorpóreas. Vet. Zootec..

[35] Dahlberg J.A., McClure S.R., Evans R.B., Reinertson E.L. (2006). Force platform evaluation of lameness severity following extracorporeal shock wave therapy in horses with unilateral forelimb lameness. J. Am. Vet. Med. Assoc..

[36] Barnes K., Faludi A., Takawira C., Aulakh K., Rademacher N., Liu C.C., Lopez M.J. (2019). Extracorporeal shock wave therapy improves short-term limb use after canine tibial plateau leveling osteotomy. Vet. Surg..

[37] Barnes K., Lanz O., Were S., Clapp K., Gilley R. (2015). Comparison of autogenous cancellous bone grafting and extracorporeal shock wave therapy on osteotomy healing in the tibial tuberosity advancement procedure in dogs. Radiographic densitometric evaluation. Vet. Comp. Orthop. Traumatol..

[38] Kieves N.R., MacKay C.S., Adducci K., Rao S., Goh C., Palmer R.H., Duerr F.M. (2015). High energy focused shock wave therapy accelerates bone healing. A blinded, prospective, randomized canine clinical trial. Vet. Comp. Orthop. Traumatol..

[39] Gallagher A., Cross A.R., Sepulveda G. (2012). The effect of shock wave therapy on patellar ligament desmitis after tibial plateau leveling osteotomy. Vet. Surg..

[40] Leeman J.J., Shaw K.K., Mison M.B., Perry J.A., Carr A., Shultz R. (2016). Extracorporeal shockwave therapy and therapeutic exercise for supraspinatus and biceps tendinopathies in 29 dogs. Vet. Rec..

[41] Dahlberg J.A., Fitch G., Evans R.B., McClure S.R., Conzemius M. (2005). The evaluation of extracorporeal shockwave therapy in naturally occurring osteoarthritis of the stifle joint in dogs. Vet. Comp. Orthop. Traumatol..

[42] Mueller M., Bockstahler B., Salicky M., Mlacnik E., Lorinson D. (2007). Effects of radial shockwave therapy on the limb function of dogs with hip osteoarthritis. Vet. Rec..

[43] Bockstahler B., Müller M., Skalicky M., Mlacnik E., Lorinson D. (2006). Die extrakorporale radiale Stoßwellentherapie bei der Cubarthrose des Hundes-eine mittels Messung von Bodenreaktionskräften evaluierte Studie. Tierärzl. Mschr..

[44] Loghlin M., Lewith G., Falkenberg T. (2013). Science, practice and mythology: A definition and examination of the implications of scientism in medicine. Health Care Anal..

[45] Simplicio C.L., Purita J., Murrell W., Santos G.S., Dos Santos R.G., Lana J. (2020). Extracorporeal shock wave therapy mechanisms in musculoskeletal regenerative medicine. J. Clin. Orthop. Trauma..

