# Supplementary material for: Systematic Review of Complementary and Alternative Veterinary Medicine in Sport and Companion Animals: Extracorporeal Shockwave Therapy
Source: Animals (Basel). 2022 Nov 12;12(22):3124. doi: 10.3390/ani12223124 (PMC9686741; doi:10.3390/ani12223124)
Supplement: Supplementary file 1 [file animals-12-03124-s001.zip › animals-1995794-supplementary.pdf]

Supplementary material

Systematic Review of Complementary and Alternative Veterinary Medicine in Sport and Companion Animals: Extracorporeal Shockwave Therapy

Anna Boström, Anna Bergh, Heli Hyytiäinen, and Kjell Asplund

Manual for assessment of risk of bias and relevance

General

- Use separate templates for controlled trials (randomized or non-randomized) and observational studies.
- Use the following grades: 0-5 or 1-5. For items with only three grades described (0,3,5), intermediate grades (1,2,4) may also be used.

Study design, controlled trials

1 = Non-randomized controlled trial, non-adequate control group
2 = Non-randomized controlled trial, adequate control group
3 = Quasirandomized trial
4 = Randomized controlled trial, randomization with flaws
5 = Randomized controlled trial, randomization without flaws

Study design, observational studies

1 = Case study
2 = Cross-sectional
3 = Case-control
4 = Retrospective cohort
5 = Prospective cohort

Statistical power

Statistical power is considered sufficient if (a) a power calculation is reported and the required number of animals were included, *or* (b) if the principal endpoint is statistically different between groups (or corresponding).

0 = Not sufficiently powered to detect an effect *or* not possible to assess
3 = Possibly sufficiently powered to detect an effect but difficult to assess
5 = Sufficiently powered to detect an effect

Confounding (observational studies only)

What impact may potential confounding (e.g. concurrent other treatment) have had on the results?

0 = severe or not reported
3 = moderate
5 = none/of marginal importance

Selection/classification (observational studies only)

How well were intervention groups defined/delineated?
Was there a risk that groups were defined after the results were known?

0 = high risk of selection/classification bias or cannot be assessed

3 = intermediate risk of selection/classification bias
5 = no risk of risk of selection/classification bias

Deviation from planned therapy
This item is assessed by weighing the answers to four questions:

- Were cross-overs or other deviations from planned therapy not reported?
- Was there substantial crossover (>10%) between intervention/control groups?
- Were there other serious deviations from planned therapies?
- If deviations occurred, how unbalanced were they between the groups?

0 = large deviations from planned therapy *or* markedly imbalanced proportions with deviations between comparison groups *or* not reported
3 = moderate deviations from planned therapy
5 = no or only minor deviations from planned therapy

Lost to follow-up

- What proportion was lost to follow-up?
- Was loss to follow-up balanced between the groups?

0 = proportion lost to follow-up >=40% or large imbalance between groups
3 = proportion lost to follow-up 20-39%, little imbalance between groups
5 = proportion lost to follow-up 0-19%, little imbalance between groups

Outcome assessment
0 = assessor aware of the group to which the animals had been assigned
3 = assessor aware of the group to which the animals had been assigned, but this probably did not have a major effect on results
5 = independent assessment of outcome (assessor unaware of treatment group, *or* laboratory, physiological, or similar measurements)

If "objective" outcomes were not used, include information on who did the assessments: the therapist (T), the animal owner (AO), a blinded assessor (BA), or other (O).

Relevance
Whereas the items used for risk-of-bias scoring are about internal validity of the study, relevance is about external validity. For the sake of simplicity, it is included here as a risk-of-bias item but may also be reported separately when the systematic reviews are compiled.

To what extent are the study results transferable to the Swedish setting?

0 = low relevance
3 = intermediate relevance
5 = high relevance

Overall assessment of risk of bias
A very serious bias in one item cannot be counterbalanced by high scores in other items. Therefore, the overall assessment of risk of bias is qualitative; an arithmetic summary score is only to be used as guidance.
The following risk-of-bias categories are used:

- Low
- Low-to-moderate

- Moderate
- Moderate-to-high
- High

References

Higgins J, Thomas J (eds,): Cochrane Handbook for Systematic Reviews of Interventions, version 6.2, 2021 [7]. https://training.cochrane.org/handbook/current.

Statens Beredning för Medicinsk och Social Utvärdering (SBU). Utvärdering av metoder i hälso- och sjukvården och insatser i socialtjänsten: en metodbok [in Swedish]. Stockholm 2020. [6] Available from: http://www.sbu.se/met. Av previous version is available in English at https://www.sbu.se/globalassets/ebm/metodbok/eng_metodboken_no-longer-in-use.pdf.
